# Isolated dissection of the superior mesenteric artery treated using open emergency surgery

**DOI:** 10.1186/1749-7922-9-47

**Published:** 2014-08-14

**Authors:** Markus Udo Wagenhäuser, Tolga Atilla Sagban, Mareike Witte, Mansur Duran, Hubert Schelzig, Alexander Oberhuber

**Affiliations:** 1 Department of Vascular and Endovascular Surgery, University Hospital Düsseldorf, Moorenstraße.5, 40225 Düsseldorf, Germany

**Keywords:** Superior mesenteric artery, Dissection, Bowel infarction, Open surgery, Aorta

## Abstract

**Background:**

Isolated dissection of the superior mesenteric artery (IDSMA) remains a rare diagnosis. However, new diagnostic means such as computed tomography makes it possible to detect even asymptomatic patients. If patients present symptomatic on admission, the risk of bowel infarction makes immediate therapy necessary. Today, endovascular techniques are often successfully used; however, open surgery remains important for special indications. In this paper, we present two cases with IDSMA and show why open surgical repair is still important in current treatment concepts.

**Methods:**

Two cases with ISDMA that presented in our department from January 1, 2014 to June 1, 2014 are described. Data collection was performed retrospectively. Additionally, a review of articles which reported small cases series on patients with IDSMA within the past five years is provided.

**Results:**

Both patients underwent open surgical repair following interdisciplinary consultation. Both patients were transferred to the intensive care unit after surgical repair and needed bowel rest, nasogastric suction and intravenous fluid therapy. CT scans were performed within the first week after operation. Platelet aggregation inhibitors were used in both cases as postoperative medication. Both patients survived and are able to participate in everyday activities.

**Conclusion:**

Open surgical repair remains important in cases of anatomic variants of visceral arteries and suspected bowel infarction. Therefore, it is important that knowledge about open surgical techniques still be taught and trained.

## Introduction

Isolated dissection of the superior mesenteric artery (IDSMA) remains a rare diagnosis; however, following the implementation of CT-scans in clinical routines, an increasing number of reports concerning patients with IDSMA can be observed [1]. The first description of IDSMA in the literature occurred in 1947 [2]. The superior mesenteric artery (SMA) is involved in over 60% of all spontaneous visceral dissections; however, its isolated dissection remains uncommon [3]. The successive course of the dissection starts with progressive thrombosis of the false lumen and continues with progressive dissection to distal branches, finally resulting in either rupture through the adventitia or the expansion of the false lumen [4,5]. A review of the literature recently reported that about 88% of all cases published to date concern men at an mean age of 54 years [6]. As abdominal pain is the most frequent sign of symptomatic IDSMA, it has been classified into grade I (peritonitis absent) and grade II (peritonitis present) [7]. The clinical course is individually different and difficult to predict. Radiological results show that angiographic follow-up findings may vary from complete remodeling to aneurysmal changes of the false lumen [8]. It can be shown that the length of the dissection correlates with the severity of abdominal pain; however, it remains uncertain whether bowel ischemia or the distention of periarterial nerve fibers is responsible for pain as a leading symptom [9]. The etiology of IDSMA is still uncertain. Cystic medial necrosis, fibromuscular dysplasia and atherosclerosis have been identified as associated with this rare disease [10]. The entry of the dissection is mostly located at the beginning of the superior mesenteric artery (SMA), i.e., about 15 mm to 30 mm of its origin, as in this area, differential forces as a result of the transition of the fixed to the mobile segment of the artery are the highest [7,10]. The latest reports show that conservative management and endovascular therapy are common therapeutic options for patients with an IDSMA today [11-13]. Open surgery is only considered if complications occur during the clinical course. In this paper, we present two cases where initial open surgery had to be performed due to abnormal vascular anatomy and a complete occlusion of the dissected SMA. The suspicion of bowel infarction prevented less invasive endovascular approaches.

## Methods

Data collection was performed retrospectively in both cases. The patients were treated in the Department of Vascular and Endovascular Surgery, Heinrich Heine University, Düsseldorf. Oral and written consent concerning the publication of medical histories and radiological findings was obtained from both patients.

Additionally, we performed a literature search to outline the increasing number of reports about patients with IDSMA during the past five years. Here, a PubMed search was performed using the keyword “superior mesenteric artery” in conjunction with the term “dissection”. We only included peer-reviewed studies that had been published between January 1, 2009 and June 1, 2014. The patient cohort of the studies had to include at least 10 patients. Results were summarized in a table and cases were subdivided based on medical treatment into “medical management”, “endovascular therapy” and “open surgery” to show the distribution of therapeutic strategies of the past five years.

## Results and discussion

### Results

#### Case 1

**Figure 1:**
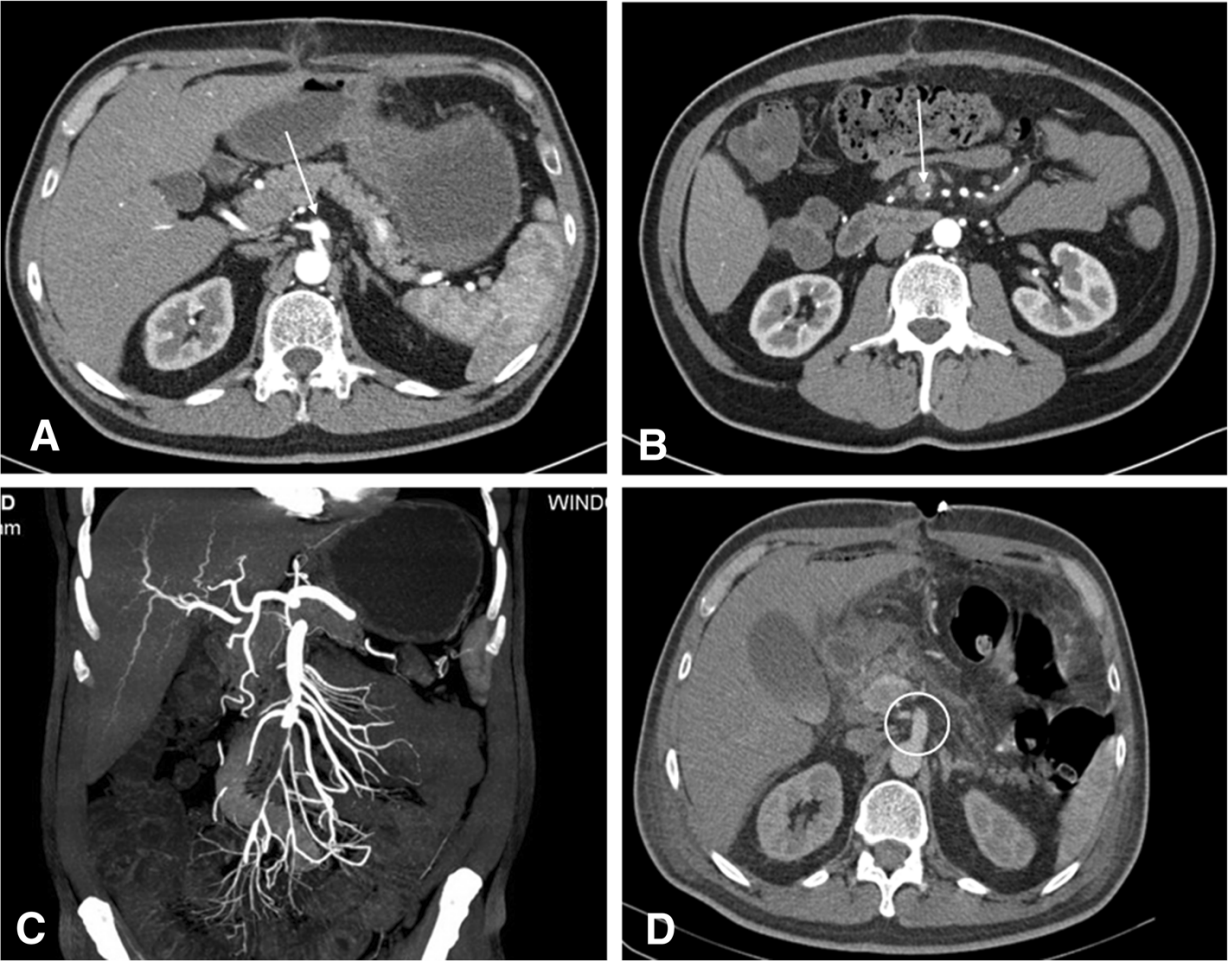
**Representative CT scan findings. A**: shown is the entry of the dissection at the proximal SMA. An abnormal origin of the right hepatic artery from the proximal SMA can be seen as an anatomical variant. **B**: An embolism of a distal branch of the SMA is shown. **C**: Reconstruction of the CT scan after admission. Almost complete decline of intestinal perfusion of the right abdominal side could be observed. **D**: findings of the control CT scan 5 days after operation. No residual membrane could be observed, normal perfusion of the SMA and the right hepatic artery.

Our report concerns a 51-year-old Caucasian man who was admitted to our clinic with severe abdominal pain. Two weeks prior, he had undergone an emergency operation in another hospital due to an IDSMA. Colleagues resected the dissection membrane and the SMA was reconstructed with a Dacron® patch. According to their medical history, the patient suffered from hypertension and non-insulin dependent diabetes mellitus (NIDDM). The patient was discharged free of symptoms two weeks prior to presentation in our department. Following admission to our emergency room, an immediate CT-scan and a blood test were performed, as the patient showed signs of an initiating peritonitis.The CT scan showed an isolated re-dissection in the proximal part of the SMA with embolization of a distal branch causing an almost complete decline of right hand side intestinal perfusion. Aggravating, the right hepatic artery originated from the proximal part of the SMA as an anatomical variant. The origin was located directly in the region of the dissection entry. Figure 1 shows the major findings of the CT scan on admission. As endovascular therapy had a high risk of post interventional liver failure, the decision for open surgery was taken at an interdisciplinary level. Blood test results showed a normal serum lactate level, while C-reactive protein (CRP) and leukocytes (WBC) were raised. Thus, the patient had to be transferred urgently to the operating theatre.We resected the dissection membrane from the origin of the SMA and a selective embolectomy of the arcade arteries was performed. The SMA was re-constructed using a venous interponate. Thus, for the interposition the saphenous vein from the right upper leg was used. The patient was admitted to the intensive care unit (ICU) with an abdomen apertum. As hypercoagulability occurred during the operation and we suspected a heparin induced thrombopenia (HIT), anticoagulation was managed using Argatroban with an activated partial thromboplastin time (aPTT) of 50-70 seconds. This suspicion was later confirmed due to a Heparin-induced Thrombocytopenia Platelet Factor 4 Antibody Test. Figure 1 demonstrates the representative findings of a CT-scan control five days after the operation. As a further course, negative wound pressure therapy was performed with wound dressing changes at intervals of two days and conducted within in the operating theatre (four times). In this context, the small intestinum was carefully inspected. We could not find any signs of hypoperfusion lesions. As the patient described persistent abdominal pain, performing a colonoscopy six days after the operation meant that ischemic colitis could be ruled out.

A secondary closure of the abdomen apertum could eventually be performed using a mesh graft transplant as coverage. Anticoagulation was managed using Fondaparinux at a therapeutic dose. After closure of the abdomen, dual platelet inhibition with clopidogrel and acetylsalicylic acid was used as a long-term medication.

Following the operation, the patient needed a bowel rest, nasogastric suction and intravenous fluid therapy. Diet was resumed after complete resolution of abdominal pain and nutritional support was required in the interval. The patient needed prokinetic medication at the outset, but during their hospital stay, a normal ingestion and defection frequency without any medical support was achieved. The patient could be mobilized and will undergo postdischarge rehabilitation.

#### Case 2

**Figure 2:**
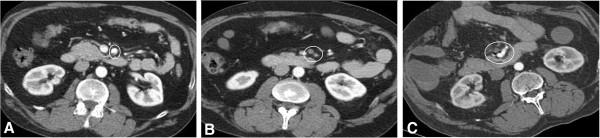
**Representative CT scan findings. A**: Dissection entry in the SMA at the typical location after passing behind the neck of the pancreas and the splenic vein. **B**: total occlusion of a branch of the SMA distal to the dissection entry. **C**: findings of the CT control 1 day after operation are shown. No residual membrane could be observed, normal perfusion of the SMA and the obstructed branch.

The second case concerns a 50-year-old Caucasian man who was admitted to our clinic after a CT scan in an external hospital indicated suspicion of an acute occlusion of the SMA. Primary CT scan findings are shown in Figure 2. The patient presented with severe abdominal pain and vomiting. On reviewing the patient's medical history, it was discovered that he had a colitis ulcerosa, first diagnosed one year previously. In September 2013, the patient underwent a sigma-resection with the creation of a descendostoma resulting from a covered perforated sigma diverticulitis. At that time, thrombosis of the inferior mesenteric vein and a branch of the portal vein could be seen and as a result, anticoagulation with Rivaroxaban was initiated and has been maintained ever since.

Initial blood tests showed elevated CRP and leukocytes, whereas serum lactate level was within normal range. Following admission to the emergency room, the interdisciplinary decision was made to transfer the patient immediately to the operation theater, as clinical symptoms made a bowel infarction likely. We resected the dissection membrane from proximal SMA to the first arcade artery. Reconstruction was done using a saphenous vein patch. Macroscopic observation showed no signs of intestinal infarction; thus, intestinal resection was not necessary. Postoperative, the patient was admitted to the ICU with an abdomen apertum. Anticoagulation was managed using intravenous heparin and an aPTT of 50-70 seconds. In due course, medication was changed to platelet inhibition with acetylsalicylic acid.A control CT scan was performed on the first day following the operation. Adequate intestinal perfusion could be seen with no signs of bowel infarction, as was verified by a second look laparotomy. Figure 2 shows the representative findings of the control CT scan. A colonoscopy five days after the operation was able to exclude intramural ischemic lesions and abdominal pain was already absent at this time. In due course, negative wound pressure therapy was performed with wound dressing changes at intervals of four days. It was possible to cover the abdomen and to bridge the fascia defect using a Vicryl mesh; thereafter, a definite closure could be performed.

Following the operation the patient needed a bowel rest, nasogastric suction and intravenous fluid therapy. We were able to initiate a light diet after the complete resolution of abdominal pain and eventually return the patient to a normal diet. The bridging of nutritional support was required. The patient could be mobilized and will perform postdischarge rehabilitation.

## Discussion

IDSMA remains a rare condition, with postmortem investigations showing an incidence of about 0.06% [14]. However, to date, an agreement on the standardized treatment for this condition has not been reached. Within the past five years, reports featuring a small series of cases of patients with IDSMA can be found in the literature; prior to this period, only case reports are predominantly available. Based on a PubMed search, we identified 14 studies that fulfill the search criteria, which consisted of 323 cases altogether. Table 1 provides an overview of these publications.

**Table 1:** Summary of small case series on patients with IDSMA

**Year of publication**	**Author**	**Total number of cases**	**Medical treatment**	**Open surgery**	**Endovascular therapy**
2014	Kim HK et al. [15]	27	27	-	-
2014	Ahn HY et al. [16]	13	12	1	0
2014	Li DL et al. [17]	42	24	7	11
2013	Dong Z et al. [7]	14	4	1	9
2013	Jia ZZ et al. [18]	17	14	0	3
2013	Li N et al. [19]	24	0	0	24
2013	Luan JY et al. [20]	18	7	0	11
2013	Choi JY et al. [21]	12	10	0	2
2012	Pang P [22]	12	3	0	9
2012	Zhang X [23]	10	6	2	2
2011	Min SI et al. [24]	14	7	1	6
2011	Park YJ et al. [25]	58	53	4	1
2011	Cho BS [26]	30	23	1	6
2009	Yun WS [9]	32	28	3	1
Sum		323	218	20	85

The investigation period was from January 1, 2009 to June 1, 2014. Cases are subdivided due to treatment strategies.

Medical treatment seems to be effective in IDSMA. During a follow-up of 18 months a reduction of occlusion in the true lumen could be seen in up to 89% and progressive resolution of false lumen thrombosis in all patients [15]. Nevertheless, a fail rate of roughly 34% among conservative therapy approaches that includes the administration of effective anticoagulation through intravenous heparin makes such an approach appear questionable [27-29].

Endovascular therapy offers safe and quick therapy for patients with IDSMA. The first description of this approach by Leung et al. was followed by multiple reports of successful treatments by several authors describing complete resolution of the pain in most cases [30-33]. In a follow-up of 6 months stent patency could be found in 100%, a false lumen patency in 22% and new development of dissection in the SMA distal to the stent in 4% of all cases [19]. Other authors reported a fail rate of endovascular interventions of up to 50% and one author even described stent misplacement in the false lumen [7,22].

Open surgery is restricted to special indications. According to the literature available validity is limited as there are little reports up to now. It seems that if open surgery is performed the risk of operative revision is up to 28.6% and mortality rate is significantly elevated compared to other therapeutic options [17]. Thus, open surgery continues to be a choice of treatment with poor prognosis for patients.

In summary, most of cases emphasize that the clinical presentation of the patient on admission should have the strongest impact on the decision-making process. Preliminary algorithms derived from this small series of cases have been introduced. Dong et al. introduced an algorithm based on a study of 14 patients. They divided the patients into symptomatic (signs of peritonitis) and asymptomatic (no signs of peritonitis) groups and suggested an intervention or emergency operation only for symptomatic manifestations. Thus, asymptomatic patients should be treated conservatively [7]. The controversial discussion concerning whether asymptomatic patients should be treated to prevent a potential intestinal infarction remains unresolved [28,30,34,35]. Another algorithm was published by Garrett Jr. et al. [6]. In this instance, operative or interventional treatment is again suggested for symptomatic patients and the procedure should depend on the morphology and location of the dissection.

Both cases presented symptomatic on admission and we suspected an intestinal infarction due to clinical presentation. Generally, we followed the above- mentioned algorithms in general; however, the first case showed the anatomic variant of an abnormal origin of the right hepatic artery, while the second case was initially suspected to be an acute embolism with signs of intestinal infarction. Therefore, both cases needed open surgical intervention and demonstrated that open surgery should still be considered as a therapeutic option if endovascular therapy is not feasible. In this instance, we agree with Katsura et al., who described three cases of IDSMA and emphasized the necessity for open surgery in the management of this disease [36]. Considering the outcome (both patients survived), bowel resection was not necessary and after rehabilitation, they could participate in normal everyday activities.

The majority of reports about IDSMA have originated from Asia. This may reflect a genetic predisposition to SMA dissection in the Asian population [8]. However, different diet habits or viral infections in the Asian population might be causal, too. None of our patients had been to Asia prior to clinical presentation.

Suzuki et al. described characteristic CT findings concerning patients with IDSMA, which included a thrombosis of the false lumen, an intimal flap, an enlarged SMA diameter, an increased attenuation of the fat around the SMA and a hematoma in the mesentery with hemorrhagic ascites [37]. A residual intimal flap could be identified in the first case, whereas the second case only showed a complete thrombosis of the lumen in the absence of any additional radiological signs. Therefore, the second case outlines that one should also consider IDSMA as a diagnosis, even though clinical and radiological signs led to the conclusion of an acute embolism as a working diagnosis.

We performed a colonoscopy to exclude an ischemic lesion in both cases within the first week following operative treatment. We believe that endoscopic endoluminal control of the intestinal mucosa provides additional patient security. We suggest considering this approach to be standardized in the postoperative therapy of patients with IDSMA, even if patients present as asymptomatic.

Both patients received effective anticoagulation during direct postoperative therapy. In due course, this was changed to antiplatelet drugs. We intend to continue this medication for at least six months, after which the patients will be seen in our outpatient department and will undergo a follow-up CT scan. This regime has been described in a retrospective analysis by Li et al. and we consider it to be reasonable [17].

## Conclusion

IDSMA remains a severe disease. Current therapeutic options suggest conservative management in asymptomatic patients, despite knowing that a failure rate of over 30% has been evidenced in such an approach [17,32]. Endovascular therapy should be the first therapeutic choice, as a hospital stay is shorter and mortality rate is lower compared to open surgery. Indications for open surgery are suspected bowel infarction or a rupture of the SMA [17]. In this paper, we presented two further cases where open surgery was performed. An anatomical variant and the suspicion of an acute embolism with bowel infarction made open surgery necessary.

## Abbreviations

IDSMA: Isolated dissection of the superior mesenteric artery; CT: Computed tomography; SMA: Superior mesenteric artery; NIDDM: Non-insulin dependent diabetes mellitus; aPTT: Activated partial thromboplastin time; ICU: Intensive care unit; CRP: C-reactive protein; HIT: Heparin induced thrombopenia.

## Competing interests

The authors declare that they have no competing interests.

## Authors’ contributions

MUW contributed substantially to the conception and design of the manuscript. He drafted the article, analyzed the data and revised them critically. TAS helped to concept the manuscript and contributed in data acquisition and interpretation. MW helped to write the article and contributed to its design. She participated in essential data interpretation. MD helped to improve the quality of the discussion as he revised this part critically. HS and AO helped to draft the manuscript. They participated in conceiving and designing the manuscript. All authors approved the final version of the manuscript.
